# MicroRNA signatures predict prognosis of patients with glioblastoma multiforme through the Cancer Genome Atlas

**DOI:** 10.18632/oncotarget.16878

**Published:** 2017-04-06

**Authors:** Ying Yuan, Hua Zhang, Xuexia Liu, Zhongming Lu, Guojun Li, Meixia Lu, Xiaofeng Tao

**Affiliations:** ^1^ Department of Radiology, Shanghai Ninth People's Hospital, Shanghai Jiao Tong University School of Medicine, Shanghai, China; ^2^ Department of Head and Neck Surgery, The University of Texas M. D. Anderson Cancer Center, Houston, TX, USA; ^3^ Department of Otolaryngology-Head and Neck Surgery, The Affiliated Yantai Yuhuangding Hospital of Qingdao University, Yantai, China; ^4^ Department of Central Laboratory, The Affiliated Yantai Yuhuangding Hospital of Qingdao University, Yantai, China; ^5^ Department of Otolaryngology-Head and Neck Surgery, Guangdong General Hospital and Guangdong Academy of Medical Sciences, Guangzhou, China; ^6^ Department of Epidemiology, The University of Texas M. D. Anderson Cancer Center, Houston, TX, USA; ^7^ Department of Epidemiology and Biostatistics and The Ministry of Education Key Laboratory of Environment and Health, School of Public Health, Tongji Medical College, Huazhong University of Science and Technology, Wuhan, China

**Keywords:** glioblastoma multiforme, microRNA, prognosis, overall survival, TCGA

## Abstract

MicroRNAs (miRNAs) play major roles in various biological processes and have been implicated in the pathogenesis and malignant progression of glioblastoma multiforme (GBM). The aim of this study was to assess the predictive values of miRNAs for overall survival (OS) of patients with GBM. MiRNA expression profiles and clinical information of 563 GBM patients were obtained from the Cancer Genome Atlas. The most significantly altered miRNAs were identified and miRNA expression profiles were performed, through principal component analysis, the least absolute shrinkage and selection operator method. The survival analysis was performed using the Cox regression models. Additionally, receiver operating characteristic (ROC) analysis was used to assess the performance of survival prediction. We used the bioinformatics tools to establish the miRNA signature for biological relevance assessment. A linear prognostic model of three miRNAs was developed and the patients were divided into high risk and low risk groups based this model. The area under the ROC curve (AUC) for the three miRNA signature predicting 5-year survival was 0.894 (95%CI, 0.789-1.000) in the testing set and0.841 (95%CI, 0.689-0.993) in all GBM patients. High risk patients had significantly shorter OS than patients with low risk (*P*< 0.001). The results from this study support a three miRNA signature for outcome prediction of GBM. These results provided a new prospect for prognostic biomarker of GBM.

## INTRODUCTION

Glioblastoma multiforme (GBM), a World Health Organization grade IV glioma, is one of the most common and aggressive primary malignancies in adults. Patients with GBM have a poor prognosis despite advances in diagnostic and therapeutic approaches and recent considerable research efforts, with a mean survival rate of approximately 3.3 % at 2 years and 1.2 % at 3 years as well as a median overall survival (OS) time of 12 to 17 months [1–3]. Besides typical genetic alterations, aberrant epigenetic mechanisms, such as DNA methylation, histone modifications, chromatin remodeling, or altered noncoding RNA expression (e.g, microRNAs), have been implicated in the pathogenesis and malignant progression of GBM [4]. Therefore, these findings have inspired the new discovery of molecularly targeted therapies as a novel approach for GBM treatment.

MicroRNAs (miRNAs) are small non-coding RNAs of 18-25 nucleotides in length that regulate gene expression by base-pairing with the 3’-untranslated region (3’-UTR) of mRNA targets, resulting in mRNA degradation and/or inhibition of mRNA translation [5]. MiRNAs play major roles in many biological processes such as cellular proliferation, apoptosis, migration, and differentiation [6–8]. To date, at least 253 and 95 miRNAs were found to be significantly upregulated and downregulated in GBM, respectively [9]. Some miRNAs have been implicated in glioblastoma development, progression and prognosis [10–12]. However, they do not necessarily reveal the similar results, due perhaps to molecular heterogeneity in tumors [13]. Moreover, there are some inconsistencies between studies due to variations in the approaches for selecting miRNAs, study design features such as small sample size, a heterogeneous admixture of cancer types, various lengths of follow-up, and data analysis without full adjustment for important prognostic parameters and treatments as well as type of array platform utilized, and the choice of control tissue. All these issues may be important sources of heterogeneity of the results reported and may bias the estimated outcomes.

By characterizing genetic and epigenetic alterations, and the expression of cancer genomes, the Cancer Genome Atlas (TCGA) project has provided a comprehensive way to understand various cancers. Prognostic miRNA signature has shown a predictive value for OS of patients with breast cancer [14, 15], colon cancer [16] and lung cancer [17] using TCGA datasets, while the similar prediction of prognosis has not performed in GBM. Since the extensive TCGA study also demonstrated a large number of miRNA expression data for GBM patients, to take advantage of utilizing this specific resource we aimed in the current study to assess the predictive value of specific miRNA signature for OS of patients with GBM from an existing large dataset of TCGA.

## RESULTS

### Selection of miRNAs with prognostic value

**Figure 1:**
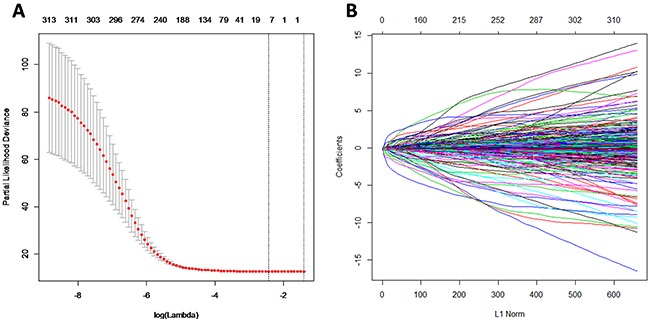
MiRNA selection using the least absolute shrinkage and selection operator (LASSO) binary logistic regression model **(A)** Tuning parameter (λ) selection in the LASSO model. The area under the receiver operating characteristic (AUC) curve was plotted versus log (λ). **(B)** LASSO coefficient profiles.

According to inclusive criteria, a total of 563 GBM patients were finally enrolled in this study. The principal component analysis included the selected 315 miRNAs with component score coefficient matrix ≥0.4 or ≤0.4 from 470 miRNAs. After the least absolute shrinkage and selection operator (LASSO) analysis, 315 miRNAs were further reduced to 9 potential predictors (Figure 1A and 1B), including hsa-miR-148a, hsa-miR-175p, hsa-miR-222, hsa-miR-302d, hsa-miR-487b, hsa-miR-608, hsa-miR-646, hsa-miR-649, and hsa-miR-675. For subsequent analysis, we randomly divided the total patients into the training set (n=282) and testing set (n=281) respectively. The mean age of patients in the training and testing groups was 58.0 and 57.9 years old, respectively. There was no significant differences of patient's age and survival time between the two sets (*P*> 0.05).

Multivariable logistic regression analyses, including following clinical candidate predictors (e.g. age, gender) and the 9 selected miRNAs (e.g., hsa-miR-148a, hsa-miR-175p, hsa-miR-222, hsa-miR-302d, hsa-miR-487b, hsa-miR-608, hsa-miR-646, hsa-miR-649, and hsa-miR-675 miRNAs), were used to evaluate the contribution of each miRNA as independent prognostic factor of patient survival in the testing set. The backward stepwise method identified four best predictors including hsa-miR-222, hsa-miR-302d, hsa-miR-646, and age (Table 1).

**Table 1:** Multivariate Cox proportional hazards analysis

Variables	Coefficient	P-value	HRs	95%CI	
				Lower	Upper
*Hsa-miR-222*	0.112	0.028	1.119	1.012	1.237
*Hsa-miR-302d*	-3.671	0.036	0.025	0.001	0.784
*Hsa-miR-646*	-2.971	0.044	0.051	0.003	0.917
Age	.023	0.000	1.023	1.013	1.033

HR, hazard ratio; CI, confidence interval.

### MiRNA prognostic model

**Figure 2:**
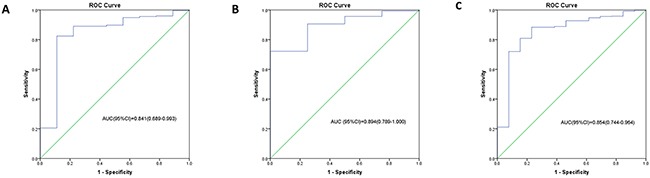
The ROC curves for the three microRNA signature in TCGA GBM cohort The ROC curve for predicting 5-year survival in GBM with an AUC of 0.841 (95%CI, 0.689-0.993) in the training set **(A)**, an AUC of 0.894 (95%CI, 0.789-1.000) in the testing set **(B)**, and an AUC of 0.854 (95%CI, 0.744-0.964) in all GBM patients **(C)**, respectively.

With coefficients from Cox regression analysis, the prognostic model was built and prognostic score was calculated as follows: Prognostic-score = (0.112×expression level of hsa-miR-222) + (-3.671×expression level of hsa-miR-302d) + (-2.971×expression level of hsa-miR-646) + (0.023×age). The miRNAs expression level was as the log2 reads per million of total aligned miRNA reads. The prognostic-score showed a great prediction of prognostic capacity for 5-year survival in GBM with an area under the curve (AUC) of 0.841 (95%CI, 0.689-0.993) in the training set (Figure 2A), an AUC of 0.894 (95%CI, 0.789-1.000) in the testing set, (Figure 2B), and an AUC of 0.854 (95%CI, 0.744-0.964) in all GBM patients (Figure 2C), respectively.

**Figure 3:**
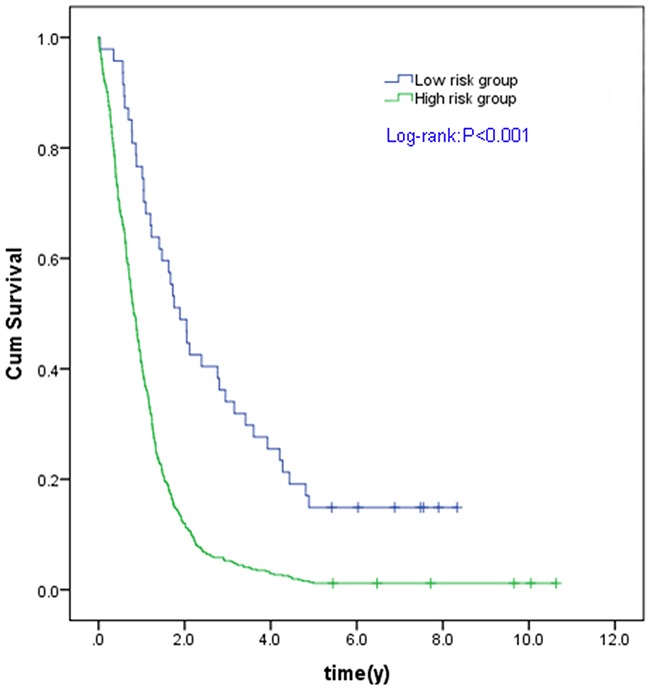
Kaplan Meier curves for the three microRNA signature in TCGA GBM cohort Individual patient was scored according to the three miRNAs signature. The Kaplan-Meier curves for GBM risk groups obtained from the TCGA cohort divided by the cut-off point. The OS of high risk group is significantly lower than that of low risk group in all GBM patients (C). The P values of the log-rank tests are <0.001.

Based on ROC curve for predicting 5-year survival in the testing set, we categorized the samples into two groups as a high risk and a low risk group using the best cutoff point of prognostic scores with optimum sensitivity and specificity. The cutoff point was -36.5428 with 90.6% sensitivity and 75.0% specificity. Compared with the patients with a low risk score, the patients with a high risk score in the TCGA GBM cohort had a significantly shorter OS (P < 0.001) (Figure 3).

### Target prediction and functional enrichment of the three miRNA signature in GBM

**Figure 4:**
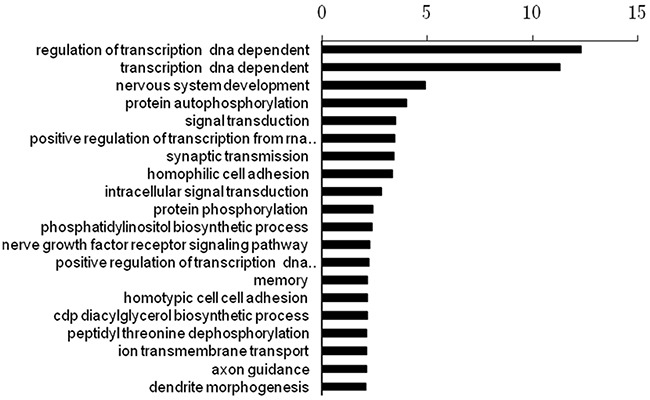
The top 20 enriched functional analysis from Gene ontology (GO) analysis

The numbers of target genes of the three miRNAs were 9017, 15613, and 9625, which were predicted by the database of miRwalk (http://zmf.umm.uni-heidelberg.de/apps/zmf/mirwalk2/). We then performed a functional enrichment analysis to elucidate the biological function of target genes of the three miRNA signature. A total of 645 pathways were enriched. The top 20 enriched functional analysis from Gene ontology (GO) analysis was shown in Figure 4. The top enriched biological process was regulation of transcription. The top enriched pathway was the Axon guidance pathway.

## DISCUSSION

In this study, we found that 3 selected miRNA signature may serve as predictor of GBM patient survival, providing novel insights into the significance of miRNAs as molecular markers in predicting the prognosis of GBM patients.

MiRNAs are regulatory nucleic acids that modulate the epigenetic state and gene expression of cells from both the tumor and its microenvironment, thereby influencing tumor cell proliferation, differentiation, survival and invasion. Alterations of miRNA expression have now been identified to be correlated with diagnosis, prognosis, and response to therapy for a range of human malignancies, including lung cancer [18, 19], breast cancer [20, 21], ovarian cancer [22] and prostate cancers [23]. Since the first two studies on investigation of miRNA expression profiles in GBM [24, 25], numerous miRNA-mediated mechanisms have been found to be implicated in the pathogenesis and malignant progression of GBM [26–28]. Dysregulation of miRNA expression level and functionality has been detected to be associated with GBM [9], although the functional properties might remain controversial or fully unknown. The pattern of miRNA expression has become a recognized tool besides other gene expression profiling for stratifying GBM patients into different groups [29]. In a previous study, a ten-miRNA expression signature was identified as survival predictors in a relatively smal sample size of 222 GBM patients [30].

Some miRNAs associated with longer or shorter survival have been identified [31]. Moreover, many of these prognostic miRNAs have validated targets and functional characteristics. In the present study, we have identified a three microRNA signature, consisting of hsa-miR-222, hsa-miR-302d and hsa-miR-646, which were validated as an independent predictor for GBM patients’ survival. The AUC of the ROC curve for the three microRNA signature predicting 5-year survival was 0.894 in the testing set and 0.854 in the total dataset, confirming a good performance for predicting survival of GBM. Among the three miRNAs, overexpression of hsa-miR-222 has been associated with poor prognosis as previously reported, and the increased expression of miR-222-3p, a target of PTEN, inhibited PTEN in glioma [32, 33]. MiR-222 is also detected to target O^6^-methylguanine methyltransferase (MGMT) mRNA. Chronic miR-222-mediated MGMT downregulation might render cells unable to repair genetic damage, leading to poor GBM prognosis [34]. Furthermore, miR-222 targeted an important cell cycle regulator of p27/KIP1 [35, 36], supporting a critical role of miR-222 in the regulation of the cell cycle, proliferation, and invasion. Suppression of miR-222 upregulated p27/ KIP1, increased the number of cells in G0/G1 stage, reduced G1/S progression, and induced apoptosis. MiR-222 also modulated the IFN-α signaling pathway including regulation of STAT1/STAT2 phosphorylation and nuclear localization [37]. An inverse relationship between pro-apoptotic PUMA gene and miR-222 expression has been demonstrated in glioma tissues [38]. MiR-222has also been shown to target intracellular adhesion molecule 1 (ICAM-1), where expression of miR-222 was inversely correlated to ICAM-1 expression in GBM tumors [39]. Microarray analysis of gene expression after inhibition of miR-221/miR-222 in GBM showed 158 differentially expressed genes involved in cell metabolism, cytoskeletal organization, and molecular signaling [37]. In a separate bioinformatics analysis, miR-221/miR-222 regulated about 70 common target genes [40]. These various targets explain the widespread regulation of miR-222 in GBM function.

On the contrary, low expression of hsa-miR-302d and hsa-miR-646 was associated with poor prognosis in patients with GBM in the current study. The miR-302 members were down-regulated in P-glycoprotein (P-gp)-overexpressing breast cancer cell lines [41], and downregulated BCRP expression to increase chemosensitivity of breast cancer cells [42]. MiR-302dmay also control proliferation and cell survival of human adipose tissue-derived mesenchymal stem cells through different targets [43]. MiR-646 is downregulated in many human cancers, and functions as a tumor suppressor. Overexpression of miR-646 inhibited lung cancer cell proliferation and metastasis, and acted as a predictor of OS [44]. Downregulation of miR-646 was also associated with progression of osteosarcoma, and played a key role in suppression of tumor in osteosarcoma [45–46]. Another study demonstrated that miR-646 may play an important role in the development and tumor metastasis of clear cell renal carcinoma [47]. However, to the best of our knowledge, this is the first to report an association of hsa-miR-302d and hsa-miR-646 with GBM.

The carcinogenic process of GBM is a multi-step process driven by a series of genetic and epigenetic alterations, resulting in the progression from a normal cell to a cancer cell. Through the enrichment and function analysis, we found that the target genes of prognostic miRNA signature may participate in many important biological processes, including regulation of transcription, nervous system development, and protein autophosphorylation.

In conclusion, we have identified a three-miRNA integrated signature that can predict the survival outcome of GBM patients. Our findings may help researchers in understanding GBM cell death and survival, developing targeted therapy, identifying high-risk GBM patients for better treatment, and warranting future evaluation of these biomarkers in patients with GBM. Modulating miRNA expression hold strong potential in therapeutic strategies and development of new molecular targeted treatment. Further understanding of molecular miRNA signatures would lead to increased survival and improved quality of life for GBM patients.

## MATERIALS AND METHODS

### Patient cohort and miRNA data

The level 3 miRNA expression profiles (level 3 data) with clinical information for GBM patients were obtained from TCGA data portal (May 2016, https://tcga-data.nci.nih.gov/tcga/tcgaHome2.jsp). Both the miRNA expression data and clinical data are open-access. To avoid the impact of unrelated causes of death, the cases with less than 1-month OS and death from other diseases or accidents were excluded in this study.

### Identification of miRNAs with prognostic value in GBM

MiRNA expression data was normalized by using the R/ Bioconductor package edgeR, which is designed for digital gene expression data [48]. The principal component analysis and LASSO method, which is suitable for the regression of high-dimensional data [49], were adopted for selection of miRNAs. The selected miRNAs were those with component score coefficient matrix ≥0.4 or ≤0.4.

Multivariable logistic regression analysis, including following clinical candidate predictors: age, gender, and selected miRNAs, was used to evaluate the contribution of each clinical variable and miRNA as independent prognostic factors of patient's survival in the testing set. The miRNA expression level was as the log2 reads per million of total aligned miRNA reads. The backward stepwise method was employed to select the best predictors. All analyses were performed using R (Packages: survival, survivalROC, boot, and superpc).

### Definition of prognostic model and ROC curve

The linear prognostic model was developed based on coefficient from Cox regression analysis. We used the linear miRNA prognostic model obtained from the training set to calculate a prognostic score for each patient. The prognostic performance was measured using time-dependent receiver operating characteristic (ROC) curves. Since the majority of events occurred before 60 months, the ability of models to predict outcome around 60 months was assessed.

In the ROC curve of the testing set for predicting 5-year survival, we chose the prognostic scores with optimum sensitivity and specificity as the cutoff values, to divide the patients into the high risk or low risk group. Kaplan-Meier curves were used to estimate the survival for patients within the two risk groups. All analyses were performed using R (Packages: survival, survivalROC, boot, and superpc). For all analyses, statistical significance was set at *p* <0.05, and all tests were two-sided.

### Target prediction and enrichment analysis

The target genes of miRNAs were predicted by miRWalk (www.umm.uni-heidelberg.de), which offers a comprehensive data of possible miRNA targets. The pathway enrichment analysis was conducted with the GeneTrail gene set enrichment tool. The results were considered significant when P value was less than 0.05 after FDR corrected.

## Abbreviations

GBM: glioblastoma multiforme
AUC: area under the curve
GO: gene ontology
LASSO: least absolute shrinkage and selection operator
miRNA: MicroRNA
OS: overall survival
ROC: receiver operating characteristic
TCGA: the Cancer Genome Atlas.
